# Association between television viewing and the risk of metabolic syndrome in a community-based population

**DOI:** 10.1186/1471-2458-8-193

**Published:** 2008-06-03

**Authors:** Pei-Chia Chang, Tsai-Chung Li, Ming-Tsang Wu, Chiu-Shong Liu, Chia-Ing Li, Ching-Chu Chen, Wen-Yuan Lin, Shin-Yuh Yang, Cheng-Chieh Lin

**Affiliations:** 1 Administration Center, China Medical University Hospital, Taichung, Taiwan; 2 Department of Medical Research, China Medical University Hospital, Taichung, Taiwan; 3 Graduate Institute of Chinese Medicine Science, College of Chinese Medicine, China Medical University, Taichung, Taiwan; 4 Biostatistics Center, China Medical University, Taichung, Taiwan; 5 Institute of Health Care Administration, College of Health Science, Asia University, Taichung, Taiwan; 6 Graduate Institute of Occupational Safety and Health, Kaohsiung Medical University, Taiwan; 7 Department of Family Medicine, China Medical University Hospital, Taichung, Taiwan; 8 Department of Family Medicine, College of Medicine, China Medical University, Taichung, Taiwan; 9 School and Graduate Institute of Health Care Administration, College of Public Health, China Medical University, Taichung, Taiwan

## Abstract

**Background:**

As a result of metabolic syndrome becoming an important issue during recent decades, many studies have explored the risk factors contributing to its development. However, less attention has been paid to the risk associated with sedentary behavior, especially television viewing. This study examined the association between television viewing time and the risk of having metabolic syndrome in a population of Taiwanese subjects.

**Methods:**

This community-based cross-sectional study included 2,353 subjects (1,144 men and 1,209 women) aged 40 and over from October, 2004 to September, 2005. Information about the time spent watching TV was obtained using a self-administered questionnaire. The definition of metabolic syndrome was according to the Third Report of the National Cholesterol Education Program's Adult Treatment Panel modified for Asians.

**Results:**

Compared to subjects who viewed TV < 14 hr/week, those who viewed TV > 20 hr/week had a 1.50-fold (95% confidence intervals (CI): 1.10, 2.03) risk for men and a 1.93-fold (95% CI: 1.37, 2.71) risk for women of having metabolic syndrome, after adjusting for physical activity and other covariates. Stratifying by the three categories of total activity levels, TV viewing time > 20 hr/week was found to still hold a significant risk for having metabolic syndrome in the lowest of the three categories of total activity level for men and in all three categories of total activity level for women.

**Conclusion:**

The findings suggest that TV viewing is an independent risk factor associated with metabolic syndrome in Taiwanese people.

## Background

There is a high worldwide prevalence of metabolic syndrome, with the rate in each country depending on the particular diagnostic definition and the ethnicity of the population. The prevalence of metabolic syndrome has been found to be 20–32 percent for Caucasians [1-5], but only 15–24 percent of the Taiwanese population has been diagnosed [6-8]. Previous studies have shown that individuals with metabolic syndrome have an increased risk to have type 2 diabetes mellitus (DM), cardiovascular disease (CVD), hypertension and stroke, etc. [9-20]. Because of its high prevalence rate among the general population and the fact that it inevitably results in type-2 DM and CVD, the syndrome has a great impact on public health.

As a result of metabolic syndrome becoming an important issue during recent decades, many studies have explored the risk factors contributing to its development. Although the syndrome has often been found to be associated with socio-demographic and lifestyle factors [5,21-26], less attention has been paid to the risk associated with sedentary behavior, especially television (TV) viewing, that has been reported to increase the risk of obesity and coronary heart disease (CHD) [27-33]. A few epidemiological studies have examined the effect of TV viewing on the chances of having metabolic syndrome and found that a prolonged viewing time of at least 2 hours per day was responsible for increasing the risk of having metabolic syndrome 1.74–3.30-fold [34-37]. However, all these studies were conducted on Caucasian populations, and it may be that Asian populations have different risk factors for metabolic syndrome. Therefore, in our study, we examined the association between TV viewing time and the presence of metabolic syndrome in a Taiwanese population as defined by the Third Report of the National Cholesterol Education Program's Adult Treatment Panel modified for Asians [38].

## Methods

### Population and participants

This is a community-based cross-sectional study. The target population consisted of people aged 40 and over who were residents in Taichung City, Taiwan, in October 2004. A total of 363,543 residents, or 4.09 percent of all Taiwanese residents aged ≥ 40 years, lived in this area during the time of our study, and the sampling frame of which was all the family records held by the Bureau of Households in Taichung City. A two-stage sampling procedure was designed to draw participants, with a sampling rate proportional to size within each stage. In the first stage of sampling, the sampling unit was Li (blocks of household units) and the selection probability for Li was set at 0.125. A total of 39 Lis were randomly selected from each city district (a total of 8 city districts). In the second stage, 4,280 individuals were selected by randomly selecting as close as 110 persons as possible from each sample Li [39-41].

Of the 4,280 subjects selected for our sample, 750 were excluded because of death (18), hospitalization or imprisonment (14), living abroad (39), moving away from the original address (411), living outside of the study area (7), not being at home during 3 attempts to visit them (202) or other reasons (59). Of the 3,530 remaining subjects, 2,359 were willing to participate in this study. However, because 6 subjects did not provide information about their TV viewing habits, 2,353 subjects (1,144 men and 1,209 women) were included in the final analysis. The age distribution among the non-participants was 47.6 percent (40–49 years), 25.5 percent (50–59 years), 13.8 percent (60–69 years) and 13.2 percent (≥ 70 years), and this was comparable with that of the participants: 43.9 percent (40–49 years), 30.9 percent (50–59 years), 14.8 percent (60–69 years) and 10.4 percent (≥ 70 years). The study was approved by the Instituted Review of Board of China Medical University Hospital. Written informed consent was obtained from all participants.

All subjects who were eligible for participation were asked to complete a self-reported questionnaire and this was followed by a series of physical examinations in the hospital (China Medical University & Hospital) between October, 2004 and September, 2005, during which participants were also required to provide a blood sample. The questionnaire required participants to provide socio-demographic characteristics and lifestyle details, such as smoking, drinking, leisure and occupational activity, and time spent on watching TV every week. Information regarding time for watching TV was obtained using the open question "On average, how many hours a day (or a week) do you spend on watching TV?" The physical activity for leisure time of a participant was determined by the different kinds of physical activities each person participated in, the average time spent on this activity per occasion and the total time per week the person spent performing each activity during the past year. The checklist of leisure-time activity contained 26 items including ball games, martial arts, aerobic sports, dancing and others. We assigned a metabolic equivalent (MET) to each kind of activity and then calculated each participant's leisure-time activity by the formula of activity: MET× averaged duration of spending on this activity (hr) × total number of times a week. The occupational activity status was determined by 4 levels according to the degree of manual labor involved: seated most of the time, light, moderate, and heavy activity and then assigned a MET to each. The occupational activity status was then calculated using the formula: manual labor intensity level × working time a day (hr) × working days a week. The adjusted carbohydrate intake by total energy intake was computed by dividing caloric intake in the form of carbohydrates by total energy intake [42], and was categorized as being either moderate (40–60 percent) or high (> 60 percent) [5].

### Anthropometric measurement and laboratory examination

Body height and weight were measured using an auto-anthropometer (Super-view, HW-666, Taipei, Taiwan) with participants wearing light clothing and no shoes. The waist circumference was measured midway between the iliac and the costal margin, with the subjects in standing position and the hip circumference was measured at its maximum in order to calculate the waist-to-hip ratio. The blood pressure was measured using participants' right arm after they had been resting in a seated position for 20 minutes. The means of two systolic and two diastolic blood pressures were recorded.

Blood was drawn from an antecubital vein in the morning after a 10-to-12-hour overnight fasting and was sent for analysis within four hours of blood. A one-spot urine specimen was also collected. Biochemical markers such as serum HDL-cholesterol, triglyceride, and fasting glucose as well as urine albumin and creatinine were analyzed using a biochemical auto-analyzer (Beckman Coulter, Lx-20, USA) at the Department of Clinical Laboratory of China Medical University Hospital.

### Definition of metabolic syndrome

A modification for Asians of the Third Report of the National Cholesterol Education Program's Adult Treatment Panel [38] was used to define metabolic syndrome. According to the report, metabolic syndrome involves three or more of the following abnormalities: a fasting plasma glucose level ≥ 110 mg/dl, a serum triglyceride level ≥ 150 mg/dl, a serum HDL-cholesterol level < 40 mg/dl for men and < 50 mg/dl for women, a blood pressure level ≥ 130/85 mmHg, or a waist circumference > 90 cm for Asian men and > 80 cm for Asian women. However, the modification is regarding the cut-offs for abdominal obesity. Subjects who took medication for hyperglycemia, hypertension, hypercholesterolemia or hypertriglyceridemia, were still considered to have abnormal laboratory data.

### Statistical analysis

Mean (standard deviation, SD) and number (percentage) of demographic characteristics and other variables categorized by status of TV viewing time status (< 14, 14–20, and > 20 hr/week) were compared using ANOVA or chi-square statistics for men and women. Four age categories were created using 10 year intervals (40–49, 50–59, 60–69, ≥ 70 yrs) while three categories were defined based on participants' education level: <9, 9–12, and > 12 yrs. Three economic status categories were created according to participants' household income per month: < 40,000, 40,000–100,000, and > 100,000 Taiwanese dollars. Smoking and drinking were categorized as never, former and current and the carbohydrate intake was either moderate or high. Leisure-time activity was dichotomized to either < 19.5 or ≥ 19.5 MET-hour per week (the first and second tertiles were combined due to only few individuals in the second tertile); the occupational activity status was categorized by the tertiles of number of metabolic equivalent hours per week: < 40, 40–83 or > 83 MET-hour per week. The total activity was obtained by summing the leisure-time and occupational activities and then divided into three categories by the tertiles: < 54, 54–103.3 or > 103.3 MET-hour per week.

Spearman correlation statistics were used to determine whether there was any correlation between certain continuous variables. Multivariate unconditional logistic regressions were used to assess the association between metabolic syndrome and the length of time spent on viewing TV (baseline: < 14 hr/week) after adjusting for other covariates. Covariates in the final models included age, level of education, household income, occupational activity status, and smoking which were significant or marginally significant in the univariate analyses.

In order to examine the joint association of total activity and TV viewing time, we examined the association between the TV viewing time and the risk of having metabolic syndrome stratified by categories of total activity in men and women.

The responses given by 241 men and 204 women indicated that they had a history of physician-diagnosed heart diseases, stroke, or cancers, but when we excluded them and re-analyzed the data, we found the results to be similar. We therefore presented our findings without exclusion. All data was analyzed using Statistical Analysis Software. Odds ratios and 95 percent confidence intervals (CI) were calculated and significance levels of p < 0.05 were reported.

## Results

Table 1 shows the socio-demographic characteristics according to groups of TV viewing time stratified by gender. In men, TV viewing time varied significantly depending on age, education level, household income, cigarette smoking, occupation activity status and their total activity status (p-value = 0.0042 to < 0.0001). For women the results were similar (p-value = 0.0004 to < 0.0001) with the exception of cigarette smoking and total activity status. The physical and biochemical variables are summarized in Table 2 according to groups of TV viewing time stratified by gender. Except for the diastolic BP in men and the serum HDL levels in both men and women, an increase in the time spent viewing TV, resulted in higher mean BMI levels, a larger waist circumference and higher systolic BP, serum fasting glucose and triglyceride levels.

**Table 1:** Distributions of sociodemographic characteristics according to status of TV viewing time stratified by gender

	**Men (n = 1144)**				**Women (n = 1209)**			
		
	**TV viewing time n (%)**				**TV viewing time n (%)**			
				
**Variables**	**< 14 (n = 329)**	**14–20 (n = 343)**	**> 20 (n = 472)**	**p-value**^b^	**< 14 (n = 342)**	**14–20 (n = 306)**	**> 20 (n = 561)**	**p-value**^b^
**Age (yrs)**^a^	56.08(0.82)	57.47(0.80)	61.15(0.68)	< .0001***	53.06(0.69)	54.46(0.73)	57.01(0.54)	< .0001***
**Education Level**				0.0027**				< .0001***
**< 9**	88(26.75)	92(26.82)	149(31.57)		120(35.09)	131(42.81)	280(49.91)	
**9–12**	141(42.86)	167(48.69)	236(50.00)		152(44.44)	129(42.16)	237(42.25)	
**> 12**	100(30.40)	84(24.49)	87(18.43)		70(20.47)	46(15.03)	44(7.84)	
**Marriage status**				0.6908				0.6338
**married**	286(86.93)	305(88.92)	418(88.56)		259(75.73)	241(78.76)	429(76.47)	
**others**	43(13.07)	38(11.08)	54(11.44)		83(24.27)	65(21.24)	132(23.53)	
**Household income (Taiwanese dollars/month)**				0.0042**				0.0002***
<**40,000**	144(43.77)	155(45.19)	260(55.08)		154(45.03)	143(46.73)	304(54.19)	
**40,000–100,000**	136(41.34)	147(42.86)	170(36.02)		138(40.35)	134(43.79)	224(39.93)	
**> 100,000**	49(14.89)	41(11.95)	42(8.90)		50(14.62)	29(9.48)	33(5.88)	
**Smoking**				0.0007***				0.3452
**never**	184(55.93)	171(49.85)	197(41.74)		327(95.61)	296(96.73)	529(94.30)	
**former**	59(17.93)	71(20.70)	132(27.97)		2(0.58)	3(0.98)	10(1.78)	
**current**	86(26.14)	101(29.45)	143(30.30)		13(3.80)	7(2.29)	22(3.92)	
**Drinking**				0.0536				0.6475
**never**	190(57.75)	188(54.81)	241(51.96)		302(88.30)	274(89.54)	496(88.41)	
**former**	36(10.94)	35(10.20)	37(7.84)		5(1.46)	1(0.33)	8(1.43)	
**current**	103(31.31)	120(34.99)	194(41.10)		35(10.23)	31(10.13)	57(10.16)	
**Adjusted carbohydrate intake**				0.6862				0.3142
**moderate**	4(1.22)	3(0.87)	3(0.64)		6(1.75)	6(1.96)	18(3.21)	
**high**	325(98.78)	340(99.13)	469(99.36)		336(98.25)	300(98.04)	543(96.79)	
**Leisure-time activity (MET-hour/week)**				0.7134				0.5496
**< 19.5**	210(63.83)	209(60.93)	298(63.14)		229(66.96)	217(70.92)	388(69.16)	
**≥ 19.5 ≤**	119(36.17)	134(39.07)	174(36.86)		113(33.04)	89(29.08)	173(30.84)	
**Occupational activity (MET-hour/week)**				< .0001***				0.0004***
**< 40**	97(29.48)	102(29.74)	208(44.07)		89(26.02)	67(21.90)	191(34.05)	
**40–83**	124(37.69)	115(33.53)	124(26.27)		138(40.35)	116(37.91)	169(30.12)	
**> 83**	108(32.83)	126(36.73)	140(29.66)		115(33.63)	123(40.20)	201(35.83)	
**Total activity status (MET-hour/week)**				< .0001***				0.1689
**0–54**	90(27.36)	105(30.61)	200(42.37)		98(28.65)	86(28.10)	192(34.22)	
**54–103.3**	120(36.47)	105(30.61)	137(29.03)		128(37.43)	103(33.66)	188(33.51)	
**103.3 <**	119(36.17)	133(38.78)	135(28.60)		116(33.92)	117(38.24)	181(32.26)	

^a^Values are expressed as means (standard deviation).

^b^For the difference among groups of TV viewing time.

*p < 0.05 (bolded);**p < 0.01 (bolded);***p < 0.001 (bolded)

MET, metabolic equivalent.

**Table 2:** Comparisons of physical and biochemical examinations among groups of TV viewing time stratified by gender

	**Men (n = 1144)**				**Women (n = 1209)**			
		
	**TV viewing time **means (standard deviation)				**TV viewing time **means (standard deviation)			
				
**Variables**	**< 14 (n = 329)**	**14–20 (n = 343)**	**> 20 (n = 472)**	**p-value**^a^	**< 14 (n = 342)**	**14–20 (n = 306)**	**> 20 (n = 561)**	**p-value**^a^
**BMI (kg/m^2^)**	24.35 (0.21)	24.76 (0.21)	25.07 (0.18)	0.0066	23.13 (0.22)	23.85 (0.24)	24.36 (0.17)	< .0001***
**Waist circumference (cm)**	84.90 (0.59)	86.11 (0.57)	87.52 (0.49)	0.0001***	74.61 (0.58)	76.52 (0.61)	78.04 (0.45)	< .0001***
**Systolic BP (mmHg)**	136.72 (1.39)	137.44 (1.36)	141.40 (1.16)	0.0022**	127.31 (1.49)	132.18 (1.57)	136.13 (1.16)	< .0001***
**Diastolic BP (mmHg)**	81.84(0.77)	82.36 (0.76)	83.26 (0.65)	0.2085	72.92 (0.81)	75.32 (0.85)	77.12 (0.63)	< .0001***
**Fasting blood glucose (mg/dl) **	103.07 (1.98)	103.63 (1.94)	110.16 (1.66)	0.0006***	97.08 (1.77)	97.81 (1.88)	104.94 (1.39)	< .0001***
**HDL-cholesterol (mg/dl)**	41.65 (0.74)	41.41 (0.72)	41.47 (0.61)	0.9548	51.22 (0.84)	49.90 (0.89)	49.81 (0.66)	0.2347
**Triglyceride (mg/dl) **	125.64 (7.52)	130.36 (7.37)	148.04 (6.28)	0.0102*	92.63 (4.61)	102.36 (4.87)	118.68 (3.60)	< .0001***

^a^For the difference among groups of TV viewing time.

*p < 0.05 (bolded); **p < 0.01 (bolded); ***p < 0.001 (bolded).

BMI, body mass index; BP, Blood pressure; HDL, high density lipoprotein.

The Spearman correlation coefficients between age, leisure-time activity, occupational activity, TV viewing time and adjusted carbohydrate intake ranged from -0.32 to 0.23, which were either weak or negligible (Table 3). The correlation between total activity with age and adjusted carbohydrate intake remained weak (Spearman correlation coefficients = -0.23 and -0.03). Therefore, there was not a strong multi-co-linearity between these covariates. On the contrary, the Spearman correlation coefficient between total activity and occupation activity was 0.92, indicating there exists collinearity between them. The Spearman correlation coefficients between TV viewing time with leisure-time activity, occupational activity, and total activities were weak (ranging from -0.04 to -0.09), implying that sedentary behavior such as TV viewing was distinct behavior from physical activity.

**Table 3:** Spearman correlation coefficients among age, variables for physical activity, TV viewing time, and adjusted carbohydrate intake.

	**Leisure-time activity**	**Occupation activity**	**Total activity**	**TV viewing time**	Adjusted **carbohydrate intake **
**Age**	0.23***	-0.32***	-0.23***	0.17***	0.06*
**Leisure-time activity**	1.00	-0.14***	0.18***	-0.04	-0.09***
**Occupation activity**	-	1.00	0.92***	-0.08*	-0.01
**Total activity**	-	-	1.00	-0.09***	-0.03
**TV viewing time**	-	-	-	1.00	-0.01
Adjusted **carbohydrate intake **	-	-	-	-	1.00

*p < 0.05 (bolded).

**p < 0.01 (bolded).

***p < 0.001 (bolded)

Compared to subjects who viewed TV < 14 hr/week, those who viewed TV > 20 hr/week, men and women respectively had a 1.50-fold (95 percent CI: 1.10, 2.03) and 1.93-fold (95 percent CI: 1.37, 2.71) chance of having metabolic syndrome, after adjusting for age, educational level, household income, occupational activity, and cigarette smoking habits (Table 4). In contrast, we found no such significant relationship when we compared the 14–20 hr/week TV viewing group with the < 14 hr/week TV viewing group. Other covariates, including age in women and cigarette smoking in both men and women, were positively and significantly associated with the risk of having metabolic syndrome. In contrast, occupational activity and educational level were negatively and significantly associated with the risk of both men and women having metabolic syndrome, after adjusting for other covariates (Table 4).

**Table 4:** Relationship between TV viewing time and other co-variates and metabolic syndrome risk by gender

	**Men (n = 1144)**				**Women (n = 1209)**			
		
**Variables**	**MS**^a ^**(n = 431)**	**Non-MS**^a ^**(n = 713)**	**Crude OR (95% CI)**	**Adjusted OR (95% CI)**	**MS**^a ^**(n = 352)**	**Non-MS**^a ^**(n = 857)**	**Crude OR (95% CI)**	**Adjusted OR (95% CI)**
**TV viewing time (hours/week)**								
**< 14**	108(32.8)	221(67.2)	1.00	1.00	66(19.3)	276(80.7)	1.00	1.00
**14–20**	111(32.4)	232(67.6)	0.98(0.71, 1.35)	0.96(0.69, 1.33)	80(26.1)	226(73.9)	1.48(1.02, 2.14)	1.38(0.93, 2.05)
**> 20**	212(44.9)	260(55.1)	1.67(1.25, 2.24)	1.50(1.10, 2.03)	206(36.7)	355(63.3)	2.43(1.76, 3.34)	1.93(1.37, 2.71)

**Age (yrs)**								
**40–49**	110(32.6)	227(67.4)	1.00	1.00	61(13.3)	397(86.7)	1.00	1.00
**50–59**	119(35.0)	221(65.0)	1.11(0.81, 1.53)	1.02(0.73, 1.42)	120(31.0)	267(69.0)	2.93(2.07, 4.13)	2.52(1.75, 3.62)
**60–69**	90(43.3)	118(56.7)	1.57(1.10, 2.25)	1.34(0.91, 1.97)	105(43.8)	135(56.2)	5.06(3.49, 7.34)	3.79(2.51, 5.72)
**≥ 70**	112(43.2)	147(56.8)	1.57(1.13, 2.20)	1.20(0.81, 1.79)	66(53.2)	58(46.8)	7.41(4.75, 11.54)	5.31(3.22, 8.77)

**Educational level**								
**< 9**	139(42.3)	190(57.7)	1.00	1.00	218(41.1)	313(58.9)	1.00	1.00
**9–12**	206(37.9)	338(62.1)	0.83(0.63, 1.10)	0.97(0.72, 1.31)	105(20.3)	413(79.7)	0.37(0.28, 0.48)	0.63(0.46, 0.86)
**> 12**	86(31.7)	185(68.3)	0.64(0.45, 0.89)	0.81(0.54, 1.19)	29(18.1)	131(81.9)	0.32(0.21, 0.49)	0.67(0.41, 1.11)

**Household income (Taiwanese thousand dollars/month)**								
**< 40**	237(42.4)	322(57.6)	1.00	1.00	214(35.6)	387(64.4)	1.00	1.00
**40–100**	149(32. 9)	304(67.1)	0.67(0.51, 0.86)	0.79(0.59, 1.06)	116(23.4)	380(76.6)	0.55(0.42, 0.72)	0.82(0.61, 1.10)
**> 100**	45(34.1)	87(65.9)	0.70(0.47, 1.05)	0.89(0.56, 1.39)	22(19.6)	90(80.4)	0.44(0.27, 0.73)	0.82(0.47, 1.43)

**Occupation activity (MET-hour)**								
**< 40**	178(41.3)	229(56.3)	1.00	1.00	122(35.2)	225(64.8)	1.00	1.00
**40–83**	137(37.7)	226(62.3)	0.78(0.58, 1.04)	0.95(0.69, 1.29)	105(24.8)	318(75.2)	0.61(0.45, 0.83)	0.94(0.67, 1.33)
**> 83**	116(31.0)	258(69.0)	0.58(0.43, 0.78)	0.64(0.46, 0.88)	125(28.5)	314(71.5)	0.73(0.54, 0.99)	1.08(0.77, 1.52)

**Cigarette smoking**								
**Never**	189(34.2)	363(65.8)	1.00	1.00	330(28.7)	822(71.3)	1.00	1.00
**former **	103(39.3)	159(60.7)	1.24(0.92, 1.69)	1.14(0.83, 1.56)	9(60.0)	6(40.0)	3.74(1.32, 10.58)	4.67(1.49, 14.69)
**Current**	139(42.1)	191(57.9)	1.40(1.06, 1.85)	1.42(1.06, 1.92)	13(31.0)	29(69.0)	1.12(0.57, 2.18)	1.35(0.66, 2.77)

^a^Values are expressed as n (percentages); OR, odds ratio; CI, confidence interval.

**Figure 1:**
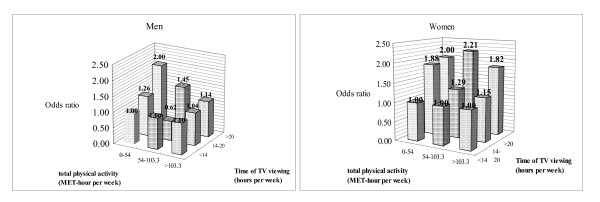
**Effect of time of television (TV) viewing and total physical activity to the risk of metabolic syndrome by gender adjusted for age, educational level, household income and cigarette smoking habits.** Groups for time of TV viewing (hours per week)was classified as < 14, 14–20, > 20 and total physical activity (MET-hr per week) as 0–54, 54–103.3, > 103.3.

When we categorized participants according to their total activity and then compared TV viewing time in each category, we found that men with TV viewing time > 20 hr/week in the lowest category of total activity had a 2.00-fold (95 percent CI:1.16, 3.46) risk of having metabolic syndrome compared to those with TV viewing time < 14 hr/week in the lowest category of total activity (Figure 1). In addition, women with TV viewing time > 20 hr/week had a significantly larger (1.82–2.21-fold) risk of having metabolic syndrome than those with TV viewing time < 14 hr/week in all three categories of total activity (Figure 1). This result indicated that there was an association between risk of having metabolic syndrome and TV viewing time in women who had sufficient physical activity.

## Discussion

In this study, we found that both men and women who on average spent more than 20 hours per week watching television had a significantly higher risk of having metabolic syndrome than those who spent less than 14 hours per week doing so. This effect was still significant, even after we adjusted for other covariates and created categories according to participants' level of total activity. To date, few epidemiological and clinical studies have examined the association between sedentary behaviors, such as TV viewing, and metabolic syndrome [34-37]. In 1995–1996, Gustat et al. (2002) conducted a community-based study in Bogalusa, Louisiana, to examine the relationship between physical activity and the risk of having insulin resistance. He recruited 409 African American and 1,011 White American participants, aged 20 to 38 years old, and used a questionnaire to measure the intensity of their physical inactivity as the sum of the time they spent watching TV and the time they spent using a computer. They divided the hours of inactivity into quintiles and found that subjects with more hours of inactivity (4^th ^and 5^th ^quintile) had a 1.9- (95 percent CI: 1.07, 3.25) and 2.5-fold respective risk (95 percent CI: 1.49, 4.19) of having insulin resistance syndrome, compared to those in the lowest quintile (1^st ^quintile) of inactivity. They concluded that the length of inactivity is an important predictor of insulin resistance syndrome in young adults. Subsequently, two other published studies that were conducted on populations of American and French subjects had similar findings [35,36]. However, these studies did not consider the interrelationship between physical inactivity, dietary habits, and metabolic syndrome.

Recently, Dunstan and his co-workers analyzed the data of 6,162 Australian people aged 35 years or older who had participated in the 1999–2000 Australian Diabetes, Obesity and Lifestyle Study [37]. Only individuals without type 2 diabetes, self-reported angina, stroke and myocardial infarction, or those taking medications for hypertension or dyslypidemia were included. They found that for each 1-hour increase in TV viewing time per day, there was a 21 percent (p = 0.07) and 26 percent (p = 0.0001) respective increase in the prevalence of metabolic syndrome in men and women, after taking into account physical activity, dietary habits, and other covariates. However, all these studies were conducted on Caucasian populations. To our knowledge, ours is the first study to observe that TV viewing is a risk factor for metabolic syndrome for an Asian population, in particular a Taiwanese population.

By which independent mechanism can the time spent viewing TV explain the association with metabolic syndrome? Firstly, TV viewing may be highly associated with poor eating habits, particularly the consumption of junk food and snacks while watching TV [43]. Although our study did not include detailed information about TV viewing and eating habits, we did have information about the daily averaged dietary intake. Even after providing for dietary intake and other covariates, we still found a significant positive association between TV viewing and the risk of having metabolic syndrome, an association that cannot be explained in full by the effect of poor eating habits while watching TV.

Secondly, previous studies have shown that there is an inverse relationship between metabolic syndrome and physical activity [34-37,44-47]. Due to modest inverse correlation between physical activity and physical inactivity (or sedentary behavior), the effect of the length time spent watching TV on the risk of having metabolic syndrome could possibly be confounded by physical activity. However, in our study, even after adjusting for occupational activity or categorized by total activity, we still found metabolic syndrome to be significantly influenced by the time spent viewing TV. This finding suggests TV viewing is an independent risk factor associated with metabolic syndrome, rather than being related to it through the effect of physical activity.

Another mechanism that could explain the effect of TV viewing on metabolic syndrome, could be that physical inactivity would reduce the triglyceride uptake from plasma to muscle and it would further lower serum HDL concentrations due to the decrease of lipoprotein lipase (LPL) activity in the skeletal muscle [48]. In this animal experiment, Hamilton slightly suspended 10 Sprague-Dawley rats' hind limbs without a weight load for 6 and 12 hours to simulate the physical inactivity. He found that the average LPL activity in rats' skeletal muscles is markedly reduced from 100 percent (SD, 7) to 59 percent (SD, 5) in 6 hours and to 12 percent (SD, 5) in 12 hours [48]. This finding supports our conclusion that the length of sedentary time (e.g., TV viewing time) is an independent risk factor for metabolic syndrome.

Even though our study population is fairly large; the study has several limitations. Our study is a cross-sectional design which makes it hard to make conclusions about the causal relationship between TV viewing time and the risk of having metabolic syndrome. Another limitation is that we did not have any information about the length of time spent using a computer and reading time, popular pastimes that also require very little physical activity. All the participants in our study were 40 years, or older and they may not be regular computer users.

## Conclusion

To summarize, we found that excess TV viewing had an adverse effect on metabolic syndrome, a finding that was found to be statistically significant. This result was not affected by physical activity. However, sufficient physical activity is associated with lower prevalence of metabolic syndrome. This implies that not only can it be reduced by an increase in physical activity but also by a decrease in sedentary behavior. A further study needs to elucidate the relationship between different kinds of sedentary behaviors and metabolic syndrome.

## Competing interests

The authors declare that they have no competing interests.

## Authors' contributions

C–CL and T–CL contributed equally to the design of the study and direction of its implementation, including supervision of the field activities, quality assurance and control. P–CC, C–SL, C–IL, W–YL, C–CC and S–YY supervise the field activities. P–CC and M–TW helped conduct the literature review and prepare the Materials and Methods and the Discussion sections of the text. P–CC, T–CL and C–IL designed the study's analytic strategy and conducted the data analysis.

## Pre-publication history

The pre-publication history for this paper can be accessed here:


